# Performance enhancement of carbonyl iron-based magnetorheological elastomers through iron-doped multi-walled carbon nanotubes reinforcement

**DOI:** 10.1038/s41598-026-36061-9

**Published:** 2026-01-21

**Authors:** Elliza Tri Maharani, Jong-Seok Oh, Seung-Bok Choi

**Affiliations:** 1 Department of Mechanical Engineering, Kongju National University, Cheonan, Chungcheongnam-do 31080 Republic of Korea; 2 Department of Future Convergence Engineering, Kongju National University, Cheonan, Chungcheongnam-do 31080 Republic of Korea; 3 Department of Future Automotive Engineering, Kongju National University, Cheonan, Chungcheongnam-do 31080 Republic of Korea; 4 Department of Mechanical Engineering, The State University of New York Korea (SUNY Korea), Incheon, 21985 Republic of Korea; 5 Department of Mechanical Engineering, Industrial University of Ho Chi Minh City, Ho Chi Minh City, 70000 Vietnam

**Keywords:** Magnetorheological elastomer, Fe-MWCNTs, Additive, Silicone rubber, Engineering, Materials science, Nanoscience and technology

## Abstract

This paper aims to explore the potential of iron-doped multi-walled carbon nanotubes (Fe-MWCNTs) as additives for enhancing the performance of magnetorheological elastomers (MREs). We investigated carbonyl iron particles (CIPs)-based MREs reinforced with Fe-MWCNTs at doping contents of 10 wt% and 50 wt%. The fabricated samples were prepared using silicone rubber as the matrix and characterized using transmission electron microscopy (TEM), high-resolution field emission scanning electron microscopy (HR-FESEM), X-ray diffraction (XRD), vibrating sample magnetometer (VSM), and rheometer. The results showed that the addition of Fe-MWCNTs enhanced the stiffness and damping performance of MREs, as the increase in storage modulus and loss modulus, respectively, especially at a current of 3 A (0.472 Tesla). Furthermore, the MRE incorporating 50 wt% Fe-MWCNTs exhibited the highest MR effect (234%), followed by the 10 wt% Fe-MWCNTs sample (220%) and the conventional CIPs-based MRE (191%). Using the conventional CIPs-based MRE (191%) as the reference, the results indicate that Fe-MWCNT doping at 50 wt% enhances the MR effect by approximately 22.5%. Our work clarifies that Fe-MWCNTs have promising potential in improving the properties of MRE for future applications in vibration-damping systems in various fields, including automotive industries, earthquake resistance, and vibration isolation.

## Introduction

A composite material is defined as a combination of two or more materials, where the resulting material exhibits enhanced properties compared to those of the individual material^1^. In various composite studies, the addition of fillers has improved mechanical, thermal properties, and wetting behavior. A previous study investigated the use of high-density polyethylene (HDPE) with nanodiamond fillers demonstrated improved characteristics for biomedical applications^2^. In another work, HDPE reinforced with multi-walled carbon nanotubes (MWCNTs) and hexagonal-Boron Nitride Nanoplatelets (h-BNNPs) showed enhancements in mechanical properties (yield stress, Young`s modulus, hardness, fracture stress, and impact toughness)^3^ and physical property (wetting behavior)^4^. Besides h-BNNPs, recycled high-density polyethylene (rHDPE) incorporating spherical-shaped silicon carbide (SiC) was also reported, giving the potential as an environmentally friendly material^5^. Furthermore, the addition of nanofillers into shape memory polymers (SMPs)^6^, which is known as shape memory polymer nanocomposite (SMPNC), could enhance mechanical and thermal properties^7^. In another work of SMPs proposed thermos-responsive polyurethane (PU) incorporating with MXene (Ti_3_C_2_T_x_) nanofiller, showing the improvements in thermal and mechanical properties for the potential applications in medical devices, sensors, robotic actuators^8^. A novel PU with structures such as rhomus, hexagon, auxetic, and their hybrids were also proposed for the application of non-pneumatic tires (NPTs) spoke^9^. A series of novel low band gap donor-acceptor for a photovoltaic device was also developed^10^.

Seeing the development and the potential of composite materials, recently, a new class of advanced composites known as magnetorheological (MR) materials has attracted significant interest for further development^11^. They are known for their unique characteristics whose mechanical properties can be easily influenced by magnetic fields. Among all MR materials, such as MR grease, MR gels, and MR plastomers, MR fluids (MRFs) are widely used for applications such as brakes, clutches, and dampers. However, MRFs have drawbacks such as poor long-term stability and magnetic particle sedimentation that can degrade their properties^12^. In addition, depending on the application of MRFs, such as an MR damper for a vehicle suspension system. This device mainly experiences leakage, which refers to undesired fluid loss from the device. Leakage can occur between the outer cylinder and piston rod, which normally uses several rings. This mainly occurs due to friction and the abrasive characteristics of carbonyl iron particles in the MRFs against sealing components^13^. The friction between MRFs and the seal surfaces causes gradual erosion and wear, which leads to seal degradation and eventually fluid leakage that decreases their viscosity and yield stress, affecting the damping performance^14^. Unlike MRFs, MR elastomers (MREs) are used as another alternative material. MREs are a kind of composite material that contains magnetic particles embedded within an elastomeric matrix. The interconnected network structure of these elastomers effectively prevents both particle leakage and sedimentation, addressing the common issues of MRFs. Additionally, in contrast to conventional rubber, MREs exhibit real-time control in mechanical properties such as stiffness and damping properties^15^, offering a wide range of applications by adjusting the magnetic field strength for vibration isolators^16,17^, actuators^18,19^, and sensing devices^20,21^.

The mechanical properties of MREs can be changed rapidly, continuously, and reversibly by using an applied magnetic field^15^. This occurs because the ferromagnetic particles align within the matrix to follow the magnetic field, altering the distribution of internal stresses and the strain energy, and resulting in a different force-displacement response^22^. To express this effect quantitatively, the magnetorheological (MR) effect is used as an indicator. It can be represented as the ratio of the absolute MR effect (the difference between the storage modulus at maximum magnetic field strength and the zero-field modulus) to the zero-field storage modulus^23^. The factors that affect the MR effect include the magnetic field strength, type and concentration of the compositions (magnetic particles and matrix), and particle distributions.

The controllable and achieved properties of MREs are obtained from their two main compositions, which are a matrix and magnetic particles. MREs can be fabricated using several types of matrices, such as natural rubber, silicone rubber, polyurethane, and thermoplastic. Meanwhile, magnetic particles of MREs include carbonyl iron particles (CIPs), cobalt particles, and nickel particles^24,25^. However, CIPs are commonly used as the magnetic particles of MREs. These particles are known as soft magnetic materials due to their high saturation magnetization and low coercivity, which are highly suitable for use in MR materials^26^. Previous studies have extensively used carbonyl iron particles (CIPs) with varying sizes^27^ and shapes^28^. To achieve the desired MR effect, the particles should be sufficiently large to accommodate multiple magnetic domains within the matrix^29^, typically with a diameter of around 1 μm to 200 μm. However, previous studies have used small-diameter magnetic particles (1–10 μm) that can improve the homogeneous dispersion within the mixture, which is essential for ensuring consistent properties of MREs. In addition, the particle shape significantly influences the properties of MREs, including MR effect, with various forms such as spherical, wires^30^, flake^31^, plate-like^32^, or flower-like^33^. Among these, spherical particles are most commonly used since they provide better dispersion stability. Therefore, achieving an optimal balance of MRE composition, such as particle shape, particle size, and dispersion uniformity, is crucial for the optimum achieved MR effect.

In MREs, additives can also be added during the fabrication process since additives play an important role in the properties of MREs. In general, additives are used as an additional composition of MREs to achieve certain properties of MREs, such as stability, flexibility, stiffness, damping, and MR effect^34^. Additive selection for MREs mainly depends on their compatibility with the matrix, which must be verified before quantifying the additive content^35^. However, the incompatibility may result in phase separation and reduced interfacial interaction of the movement of particles within the matrix. Among the various additives, silicone oil is one of the common additives that acts as a plasticizer in MRE and is used as a softening agent for decreasing the storage modulus and enhancing the MR effect^36,37^. In addition, several works have used particles such as carbon-based particles for MRE additives. Carbon black additive was first introduced in 2008 by Chen et al.^38^. Anisotropic MREs based on natural rubber were fabricated with varying concentrations of carbon black to improve their mechanical performance. It gave an MR effect improvement reaching 104% by adding 7 wt% carbon black. Meanwhile, the isotropic silicone rubber-based MREs with carbon black showed a slight increase in MR effect by 22%^39^. Another study by Jung et al.^40^ fabricated anisotropic natural rubber-based MREs with 7.4 wt% carbon black achieved the MR effect only up to 21%.

Another type of carbon-based additive is nanoparticles, such as carbon nanotubes (CNTs) that are smaller than 100 nm, offering advantages such as a high surface-to-mass ratio, high aspect ratio, and lightweight. Li et al.^41^ reported that adding 1 wt% pure MWCNTs to anisotropic MREs enhanced their stiffness (30%), damping (40%), and MR effect (70%). Aziz et al.^42^ investigated MR elastomers containing different functionalized types of MWCNTs, which were carboxylic multi-walled carbon nanotubes (COOH-MWCNTs) and hydroxylic multi-walled carbon nanotubes (OH-MWCNTs). The studies found that the addition of 1 wt% functionalized MWCNTs improved storage modulus and MR effect only up to 17.5%. However, the addition of MWCNTs into MREs resulted in a lower loss factor compared to MREs without MWCNTs. The loss factor peaks for all MWCNT-containing samples were slightly lower and nearly the same, suggesting no significant improvement in damping capability. This was attributed to the fact that MWCNTs are non-magnetic materials. Consequently, the mobility of rubber macromolecular chains is significantly restricted, leading to reduced energy dissipation and lower damping characteristics. Furthermore, the excessive additive composition more than 1.5 wt% of COOH-MWCNTs as the additive of MREs reduced the magneto-induced modulus and MR effect since CIPs within the matrix had difficulty aligning into chain-like structures along the direction of the applied magnetic field^43^.

Despite the efforts to introduce various types of additives, there remain limitations in the performance improvements of MREs, as discussed above. To address this issue, we propose carbonyl iron particle (CIP)-based MREs filled with iron-doped multi-walled carbon nanotubes (Fe-MWCNTs) as a promising additive. Unlike other carbon-based additives, Fe-MWCNTs offer not only the typical mechanical reinforcement but also additional magnetic interactions due to the presence of iron nanoparticles^44,45^. These particles are expected to enhance the alignment and mobility of fillers under an external magnetic field, thereby strengthening the interfacial bonding between the fillers (CIPs and Fe-MWCNTs) and the elastomer matrix. Such interactions are particularly important in MR materials, where field-responsive behavior strongly depends on the dynamic response of the dispersed particles. Therefore, Fe-MWCNTs can potentially overcome the common drawbacks observed in previous MRE additives, such as restricted particle mobility and weak filler–matrix interactions.

In this context, the selection of Fe-MWCNTs as an additive is motivated by their dual functionality: they provide mechanical reinforcement through the MWCNT while also simultaneously contributing magnetic responsiveness from the doped iron particles. This combined effect is expected to enhance both the rheological properties and MR effect of MREs, which are crucial for vibration and adaptive engineering applications. The originality of the present work lies in the fact that, unlike conventional CNTs or functionalized MWCNTs, the reinforcement of Fe-MWCNTs into CIP-based MREs has not yet been systematically investigated. Therefore, the objective of this study is to fabricate CIP-based MREs with Fe-MWCNTs and to comprehensively evaluate their microstructural characteristics, crystallinity, magnetic behavior, and rheological performance under varying magnetic fields. By clarifying these unique properties, this work aims to establish Fe-MWCNTs as an effective additive for the development of advanced MRE compositions in practical engineering applications.

## Materials

Spherical shape CIPs type C3518 with diameters around 3–5 μm, as magnetic particles, were purchased from Sigma Aldrich. These magnetic particles are commonly used as MRE fillers, which typically have an average particle size distribution of d50^46^. As additives, Fe-MWCNTs with doping contents of 10 wt% and 50 wt% were obtained from Korea Nanomaterials. The doping contents of 10 wt% and 50 wt% Fe on MWCNTs were selected in this work to represent two distinct doping types. The lower doping (10 wt%) and higher doping (50 wt%) were used to examine the extent to which the doping content could influence the rheological behavior of the MRE. These two concentrations would provide a clear comparison of MREs’ properties. The Fe-MWCNTs have an outer diameter of over 55 nm, an inner diameter of 8 nm, and a length of around 10–30 μm. Meanwhile, the spherical shape iron (Fe) nanoparticles for MWCNTs doping have an average size of 25 nm. The matrix material used was silicone rubber (Ecoflex 00–20), composed of two components, Part A and Part B.

### Sample preparation and test procedure

#### Fe-MWCNTs dispersion

**Fig. 1:**
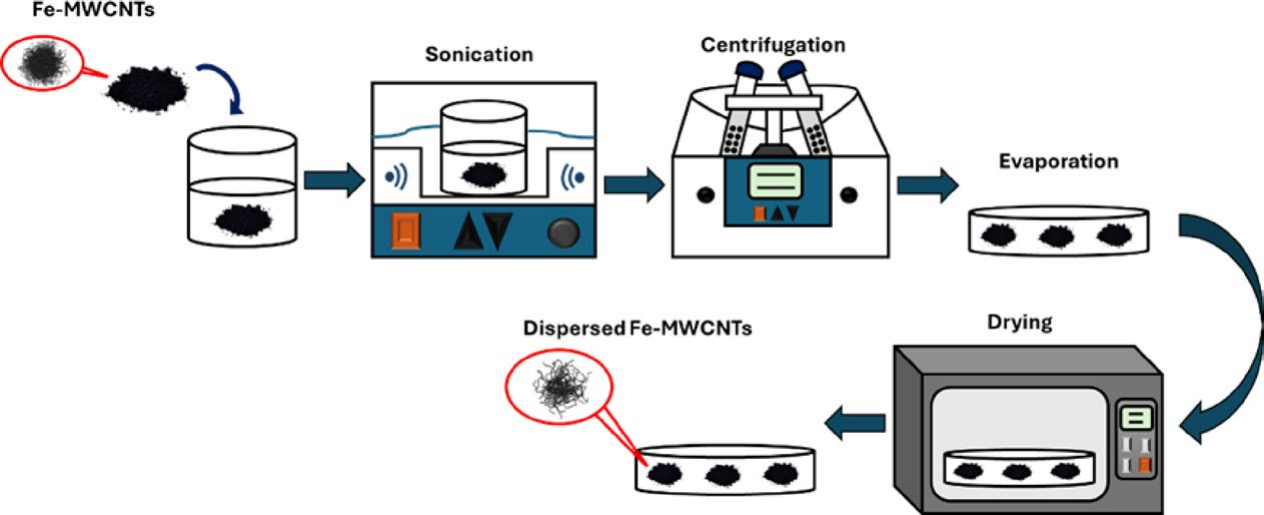
Fe-MWCNTs dispersion.

The dispersion process, as seen in Fig.1, was done to prevent the agglomeration and break the Van der Waals interaction of Fe-MWCNTs^47^. Initially, the Fe-MWCNTs were sonicated in ethanol for 6 h, followed by centrifugation at 4000 rpm for 30 min to separate the dispersed powder. Subsequently, the obtained Fe-MWCNTs powder was evaporated to remove any remaining solvent and dried in a vacuum oven at 60 °C for 24 h.

### MREs sample fabrication

**Fig. 2:**
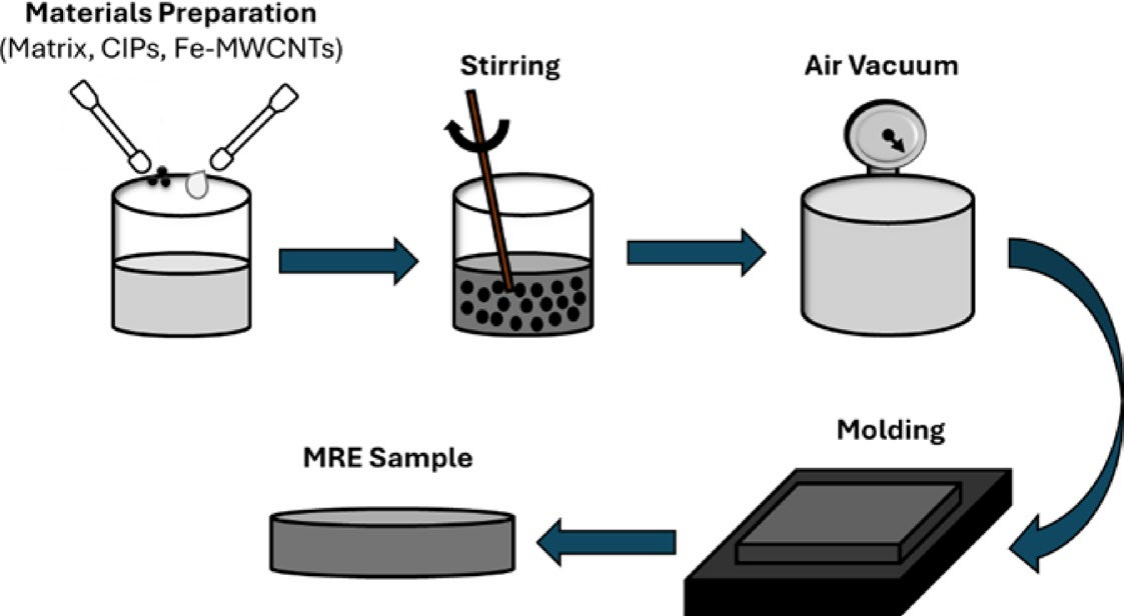
MREs fabrication.

MREs were fabricated with a composition of 60 wt% CIPs, 1 wt% Fe-MWCNTs, and silicone rubber parts A and B in a 1:1 ratio mixture. As seen in Fig. 2, the components were thoroughly mixed for 10 min to obtain a homogeneous mixture. In this step, ensuring a homogeneous mixture is important since the uniform distribution of magnetic particles and additives within the matrix influences the crosslinking behavior. The mixture was observed visually to confirm the absence of clumps, agglomeration, or noticeable color difference that could indicate poor dispersion. An air vacuum process was then conducted to eliminate any trapped air or voids before the mixture was poured into a mold. The curing was done at room temperature for 4 h.

The MRE samples were then prepared for testing purposes with a diameter of 20 mm and a thickness of 1 mm for rheological properties measurement using a rheometer and morphological analysis using high resolution field emission scanning microscope (HR-FESEM). In addition, MRE samples were also prepared with a 6 mm diameter and 1 mm in thickness for the measurement of a vibrating sample magnetometer (VSM).

### Transmission electron microscopy (TEM)

The observation of morphological structure and elemental composition of the Fe-MWCNTs was done using transmission electron microscopy (TEM, Jeol JEM-F200) equipped with energy dispersive X-ray spectroscopy (EDS), with the accelerating voltage of operation at 200 kV, with the magnification of 15,000×.

### X-ray diffraction (XRD)

The crystallinity of the fillers (CIPs, Fe-MWCNTs 10 wt%, Fe-MWCNTs 50 wt%) was analyzed using an X-ray diffraction (XRD, Rigaku MiniFlex600). The measurements were carried out with copper (Cu) as the radiation target over a 2θ range of 20° to 70°, under operating conditions of 15 mA and 40 kV.

### High-resolution field emission scanning microscope (HR-FESEM)

The morphological and elemental analysis of the MRE sample was observed using a high-resolution field emission scanning electron microscope (HR-FESEM, Tescan MIRA3-LMH) with an energy dispersive X-ray spectroscopy (EDS) that was operated up to 20 kV and was taken at magnifications of 2,000× and 15,000×. The samples were coated with platinum to improve image clarity.

### Vibrating sample magnetometer (VSM)

The magnetic properties of the particles (CIPs and Fe-MWCNTs) and MRE samples were measured using a vibrating sample magnetometer (VSM, Lake Shore Cryotronics 8604). The samples were mounted in a holder specifically designed for the test. The measurements were conducted at room temperature with the magnetic field gradually increasing to 15,000 Oe.

### Rheometer

**Fig. 3:**
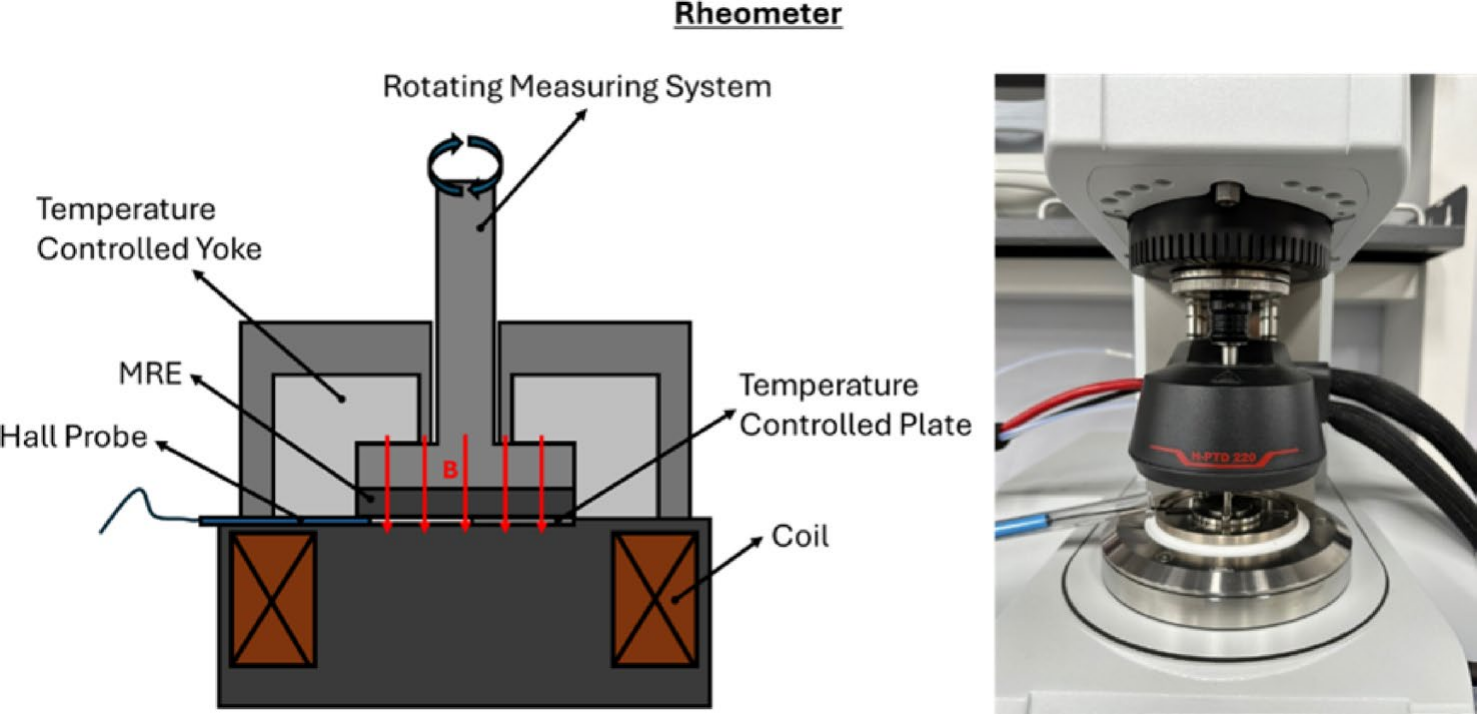
Rheometer.

The rheological measurements of the MREs were conducted using a rheometer (Anton Paar, Physica MCR 302), illustrated in Fig. 3. The setup included an MR device (MRD 170/1 T) and a profiled parallel plate measuring system (PP20/MRD/T1/P2). An electromagnetic coil beneath the parallel plate generates a magnetic field strength that can be controlled using the RheoCompass 1.34 control panel software. A teslameter (FH 54) was also utilized between the parallel plate and rotating measuring system to monitor and record the generated magnetic field connected to the software. Each testing condition of the rheological measurements was conducted three times to ensure the results obtained are accurate.

In our study, a pre-load of 15 N was applied before testing to prevent the sample from slipping on the plate. This pre-load was also used as testing preparation in another studies^48,49^. The applied axial pre-load may not cause significant deformation of the sample compared with the strains imposed during shear loading. As reported in recent studies^50^, increasing the static normal force mainly improves contact between the sample and plates, even though wall slip may still occur. A rheometer instrument commonly has a static axial pre-load up to 50 N, at which point sample fracture may occur. Regarding this, visual access to the sample during measurement is not possible due to the geometric constraints of commercial magneto-coupled rheometer configurations, particularly the two-part yoke enclosure. However, the pre-load only ensures stable positioning without introducing measurable bulk deformation. During the oscillatory shear test, the strain amplitudes applied are substantially larger than any possible compression from the pre-load. Therefore, the pre-load induced deformation is negligible relative to the shear strain range in this work and does not influence the nonlinear response such as the intrinsic viscoelastic or magneto-mechanical properties of the MREs. Other than pre-load set up, the test was conducted at 25 °C temperature in oscillatory mode by varying amplitude and frequency at off-state condition without current input (0 A) and on-state condition at a current input of 3 A (0.472 Tesla). An amplitude sweep was performed with shear strains from 0.001% to 10% at a constant frequency of 1 Hz to determine the linear viscoelastic region (LVE) of the MR elastomer. Meanwhile, a frequency sweep was conducted to analyze the dynamic properties of the elastomer by applying frequencies from 0.1 Hz to 100 Hz at a constant shear strain of 0.02%. Since MREs are widely used for various kinds of applications, including dampers and mounts, the operating conditions require slow vibrations to higher damping frequencies. Therefore, the frequency of 0.1 to 100 Hz was used to evaluate the dynamic behavior from slow oscillation (0.1 Hz) to faster oscillation (100 Hz) in order to characterize the stiffness (storage modulus) and energy dissipation as a damping property (loss modulus).

The evaluation of the changes in the storage modulus by applying a constant amplitude of 0.02% and frequency of 1 Hz while varying the magnetic flux density from 0 Tesla to around 0.7 Tesla (magnetic field sweep) was conducted. The results of the magnetic field sweep can be used to calculate the relative MR effect of the samples, as expressed in Eq. (1)^51–54^.

1 $$\:MR\:Effect=\:\frac{\varDelta\:G}{{G}_{0}}\times\:100\mathrm{\%}=\frac{{G}_{max}-{G}_{0}}{{G}_{0}}\times\:100\mathrm{\%}$$

where $\:{G}_{0}\:$is the storage modulus when no magnetic field is applied, while $\:{G}_{max}$ is the value of the storage modulus when the magnetic field reaches its highest strength.

## Results

### Morphology of MWCNTs

**Fig. 4:**
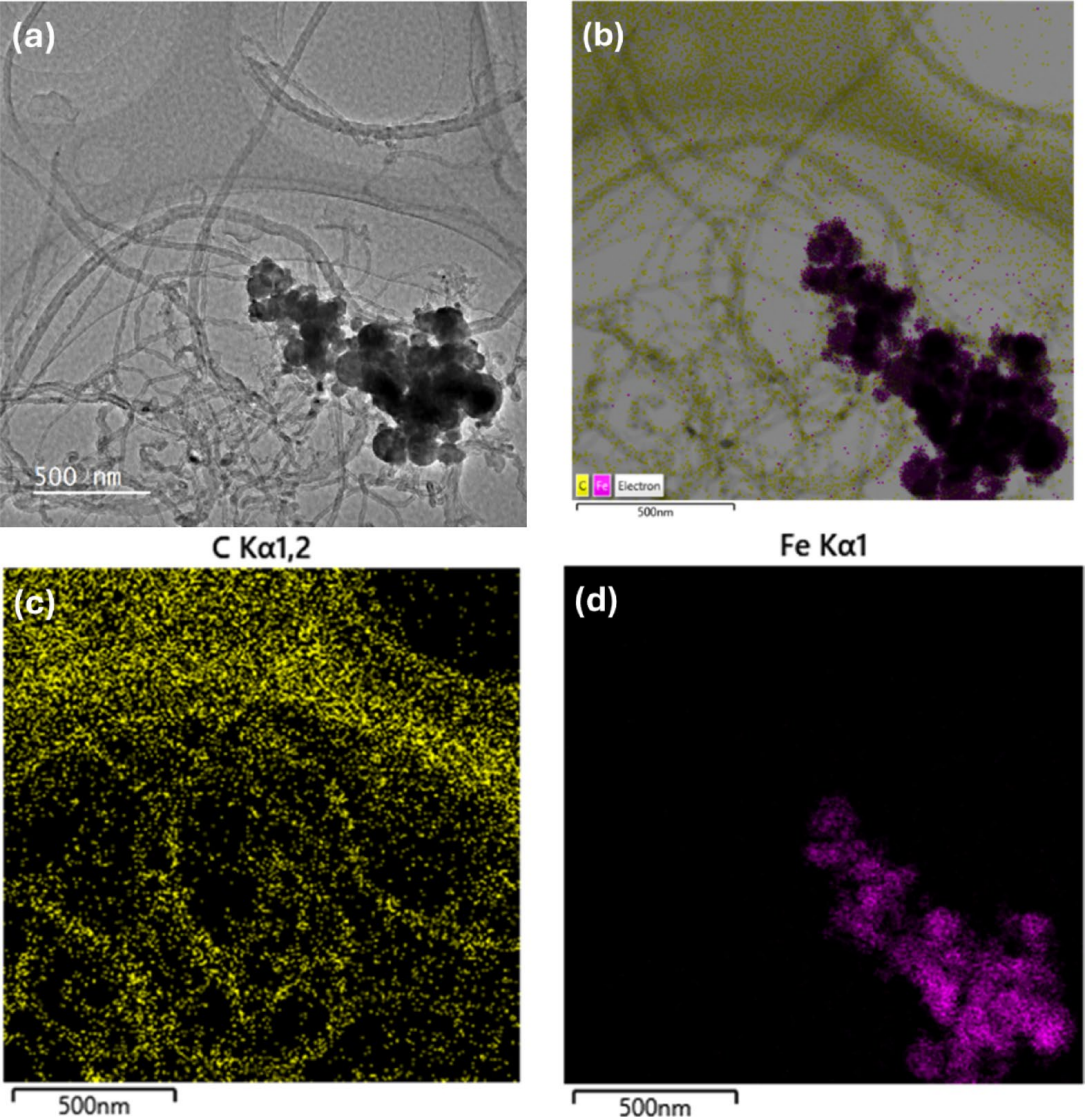
Structure and composition of Fe-MWCNTs 10 wt%. (**a**) TEM images. (**b**) Overall elemental distribution of EDS mapping. (**c**) Elemental distribution of C in the Fe-MWCNTs. (**d**) Elemental distribution of Fe in the Fe-MWCNTs.

**Fig. 5:**
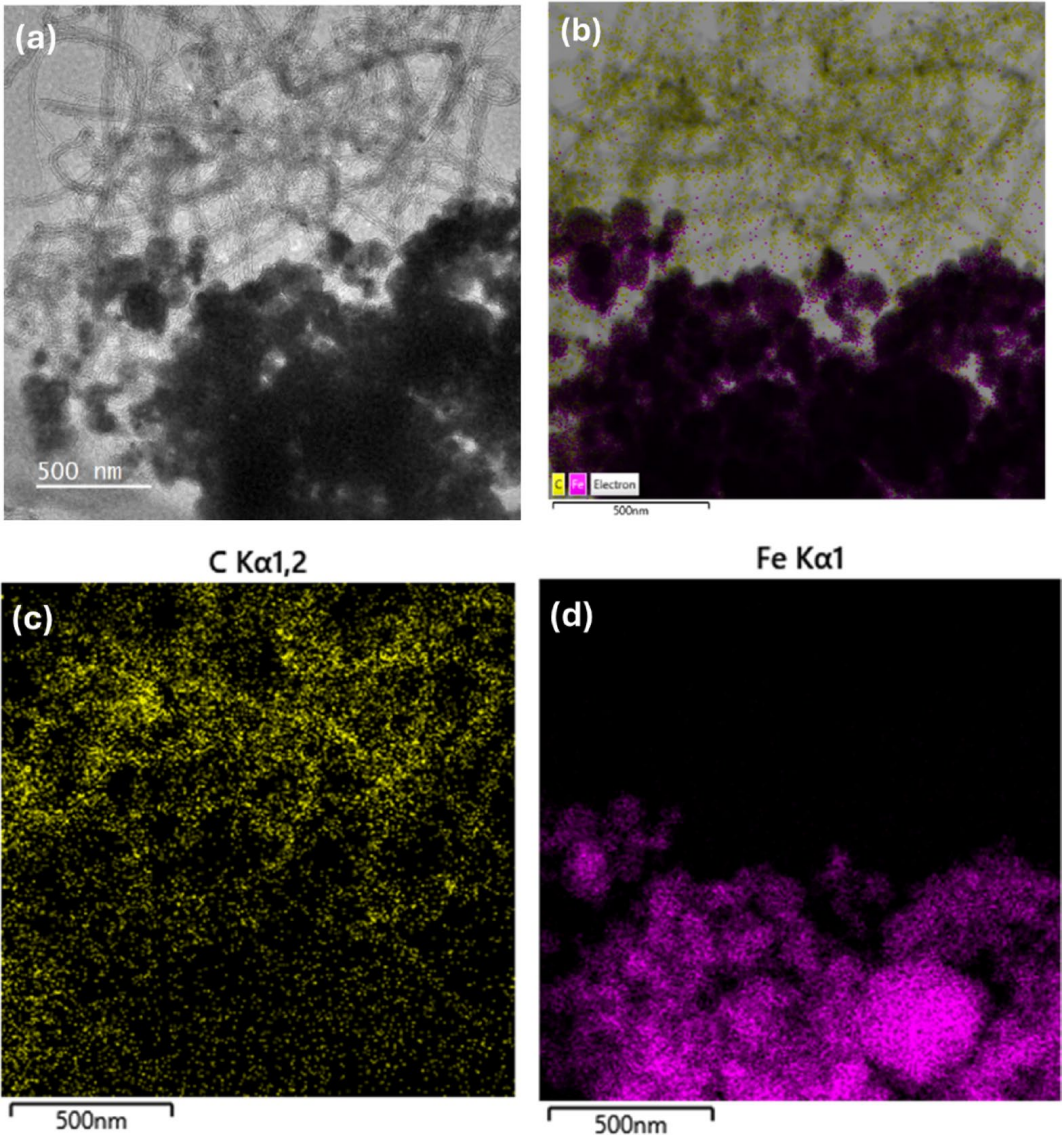
Structure and composition of Fe-MWCNTs 50 wt%. (**a**) TEM images. (**b**) Overall elemental distribution of EDS mapping. (**c**) Elemental distribution of C in the Fe-MWCNTs. (**d**) Elemental distribution of Fe in the Fe-MWCNTs.

The morphological structure and elemental composition of the materials (Fe-MWCNTs 10 wt% and Fe-MWCNTs 50 wt%) incorporated into the proposed MRE were investigated through TEM observation to confirm their suitability as the additives and functional components. The TEM images and EDS mapping of Fe-MWCNTs 10 wt% and Fe-MWCNTs 50 wt% are shown in Figs. 4 and 5, respectively. The nanoparticles showed hollow rod-like morphology and spherical shapes as MWCNTs and Fe-doping morphological structures, respectively. There was no major structural damage, indicating the doping process did not influence their structural integrity. The individual MWCNTs tended to intertwine with each other and showed interconnected structures. Even though the dispersion process was conducted, both Fe-MWCNTs 10 wt% and Fe-MWCNTs 50 wt% may have some remaining entangled structures as seen in Figs. 4a and 5a, respectively. This is most likely caused by the strong Van der Waals interactions and high aspect ratio. These interactions lead to the natural aggregation and bundling of the Fe-MWCNTs, making complete separation challenging even when using an ultrasonication method^47^. Based on the results of the EDS mapping analysis, as shown in Fig. 4b, c,d, the Fe-MWCNTs sample with 10 wt% iron doping exhibited a higher C composition compared to Fe. This suggests that the composition of Fe in this sample was relatively low. In contrast, the Fe-MWCNTs sample with 50 wt% Fe doping showed a significantly higher Fe composition as depicted in Fig. 5b, c,d, indicating more iron that was attached to the surface of MWCNTs. This comparison demonstrates that as the Fe doping increases, the composition of Fe detected by EDS also rises accordingly.

### XRD analysis of the fillers

**Fig. 6:**
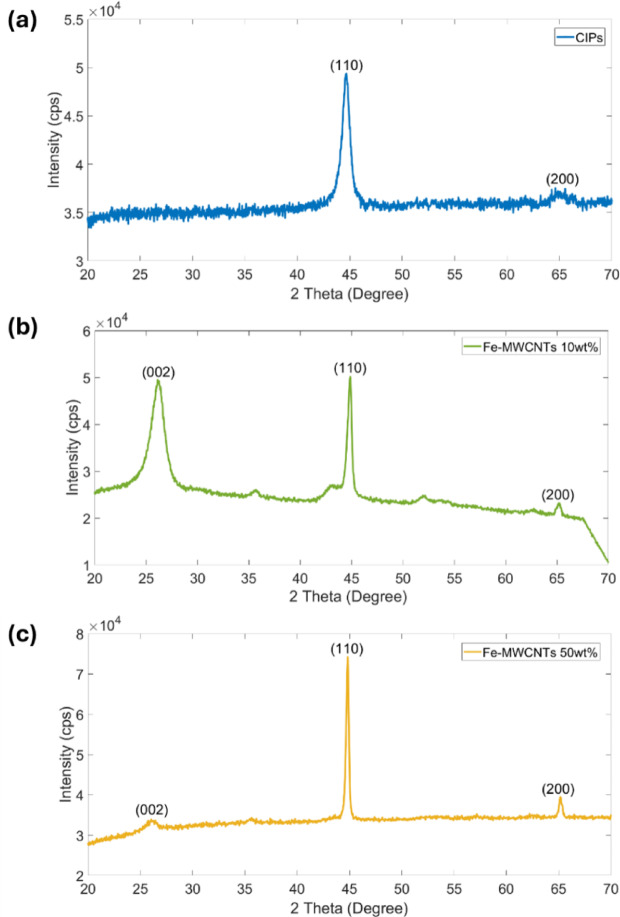
XRD pattern of the fillers. (**a**) CIPs. (**b**) Fe-MWCNTs 10 wt%. (**c**) Fe-MWCNTs 50 wt%.

Figure 6 presents the XRD pattern of CIPs, Fe-MWCNTs 10 wt%, and Fe-MWCNTs 50 wt%. As illustrated Fig. 6a, CIPs exhibited a well-defined crystal structure corresponding with the PDF: Fe 00-006-0696, showing characteristic diffraction peaks at 44.62° (110) and 64.62° (200). Meanwhile, the XRD pattern of Fe-MWCNTs 10 wt% revealed diffraction peaks at 26.12° (002), 44.88° (110), 65.16° (200) that can be seen in Fig. 6b. A similar XRD pattern was also observed in Fe-MWCNTs 50 wt% as seen in Fig. 6c, showing the diffraction peaks at 26.04° (002), 44.84° (110), 65.16° (200). Both samples showed good agreement with JCPDS#87-0722^55^. The Fe was assigned to lattice planes of (110) and (200), while graphite was indexed to the (002) lattice plane^56^.

### Magnetic properties of the fillers

**Fig. 7:**
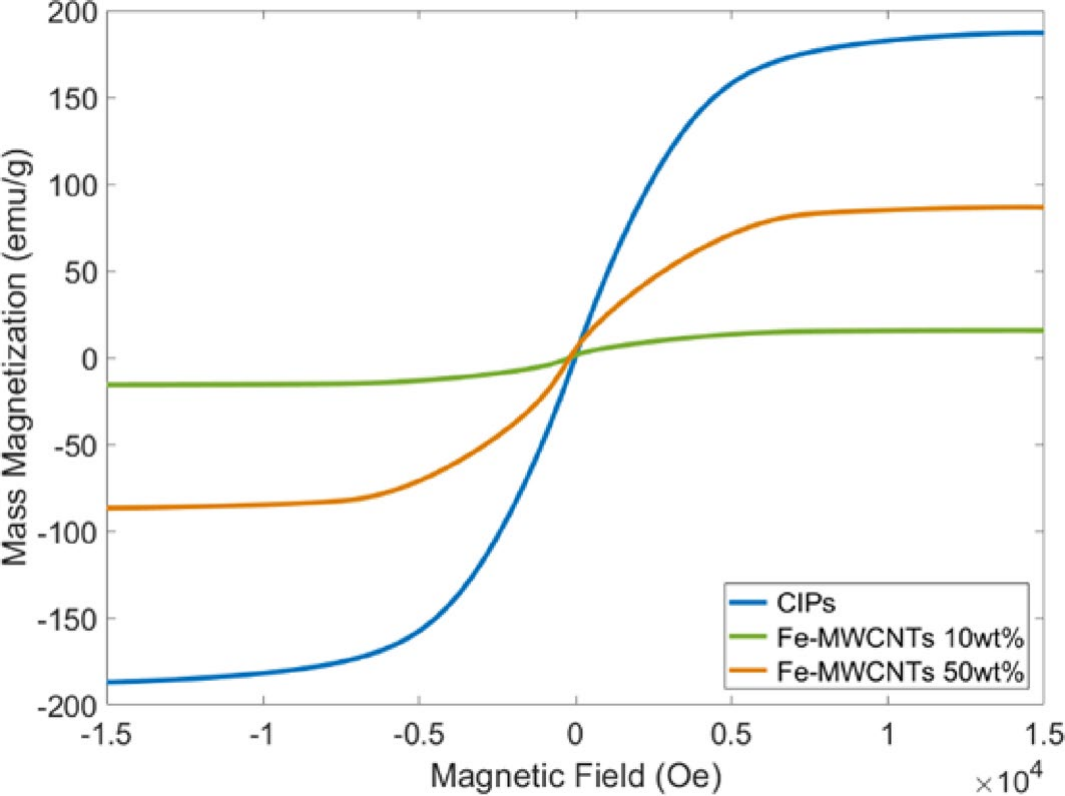
Magnetic properties of fillers.

Figure 7 shows the measured magnetic properties of CIPs, Fe-MWCNTs 10 wt%, and Fe-MWCNTs 50 wt%. The detailed values of the magnetic properties are presented in Table 1 to investigate their behavior under varying applied magnetic field strengths. CIPs showed the highest saturation magnetization of 186.90 emu/g, indicating a strong magnetic response due to the high content of pure magnetic material. Interestingly, Fe-MWCNTs 10 wt% and Fe-MWCTNs 50 wt% had saturation magnetization of 15.58 emu/g and 86.43 emu/g, respectively, reflecting the presence of Fe doping on the MWCNTs. Fe-MWCNTs had a higher remanent magnetization than CIPs. The 50 wt% Fe-MWCNTs sample showed a remanent magnetization of 4.93 emu/g, significantly exceeding the 0.63 emu/g observed in CIPs. Furthermore, the coercivity of Fe-MWCNTs 10 wt% (162.35 Oe) and Fe-MWCNTs 50 wt% (182.36 Oe) is notably higher than that of CIPs (12.17 Oe). Therefore, the compatibility of the mixture between CIPs and Fe-MWCNTs after being embedded into the silicone rubber matrix was also measured using VSM, which is presented in the next subsection.

### Morphology of MRE

**Fig. 8:**
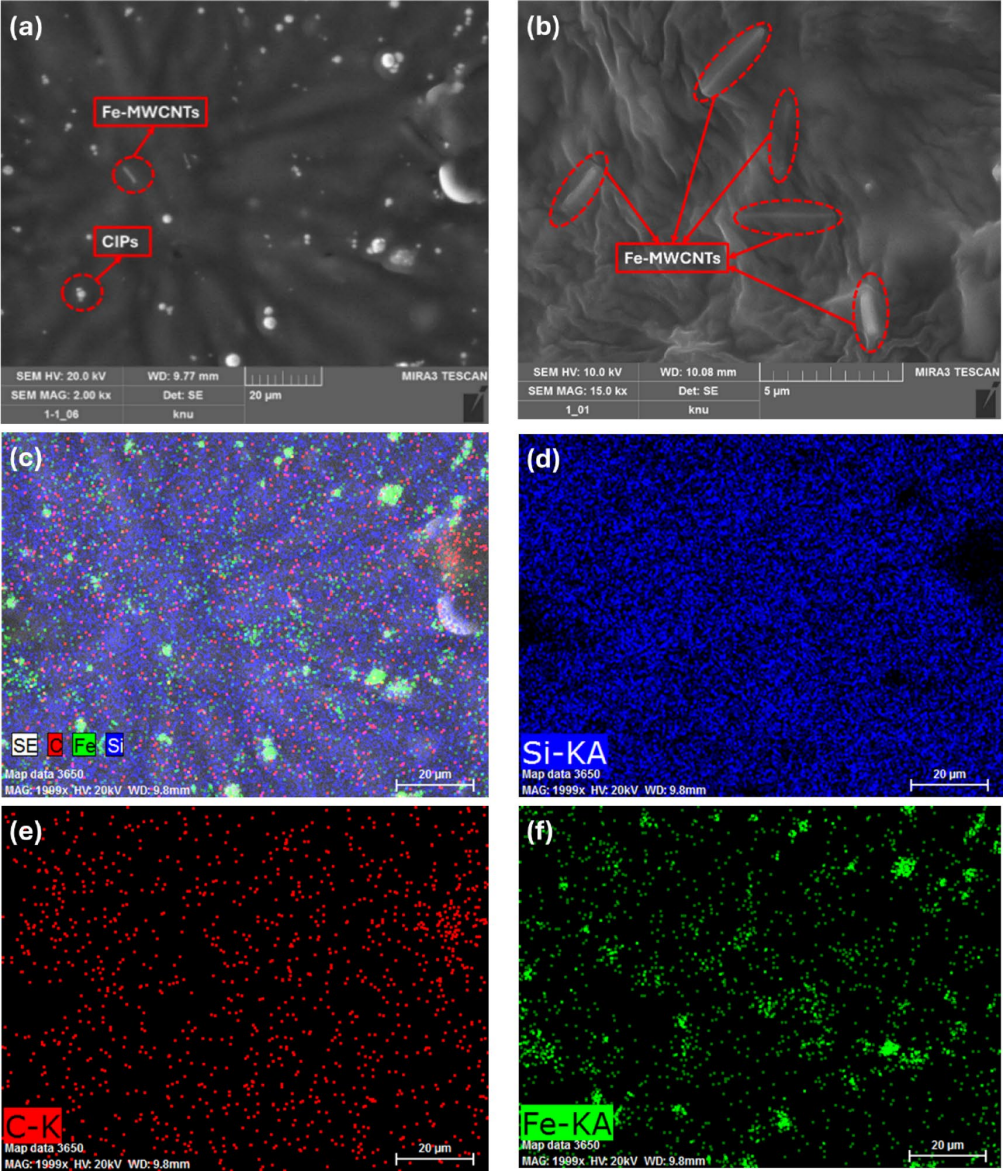
Structure and composition of the MRE CIPs + MWCNTs 50 wt%, showing the distribution of CIPs and Fe-MWCNTs in the silicone rubber matrix. (**a**) HR-FESEM image of 2,000*×* magnification. (**b**) HR-FESEM image of 15,000*×* magnification for Fe-MWCNTs observation. (**c**) Overall elemental distribution of EDS mapping. (**d**) Elemental distribution of Si in the MRE. (**e**) Elemental distribution of C in the MRE. (**f**) Elemental distribution of Fe in the MRE.

The dispersion of CIPs and Fe-MWCNTs 50 wt% within the matrix was observed using HR-FESEM, which can be seen in Fig. 8. As seen in Fig. 8a, which was taken at 2,000× magnification, the microstructure and dispersion of the CIPs (spherical shape) and Fe-MWCNTs (rod-like shape) were found within the matrix. The clear image of the morphological characteristics of MRE showing the uniform and well-dispersed distribution of Fe-MWCNTs could be observed at the magnification of 15,000× (Fig. 8b). A similar morphological shape (rod-like shape) of MWCNTs within a polycarbonate composite material was also observed using FESEM^57^. The distribution of CIPs and Fe-MWCNTs throughout the silicone rubber matrix was also identified using EDS mapping, which is shown in Fig. 8c–f.

**Table 1:** Magnetic properties of fillers.

Materials	Magnetic saturation (emu/g)	Remanent magnetization (emu/g)	Coercivity (Oe)
CIPs	186.90	0.63	12.17
Fe-MWCNTs 10 wt%	15.58	1.28	162.35
Fe-MWCNTs 50 wt%	86.43	4.93	182.36

### Magnetic properties of MREs

**Fig. 9:**
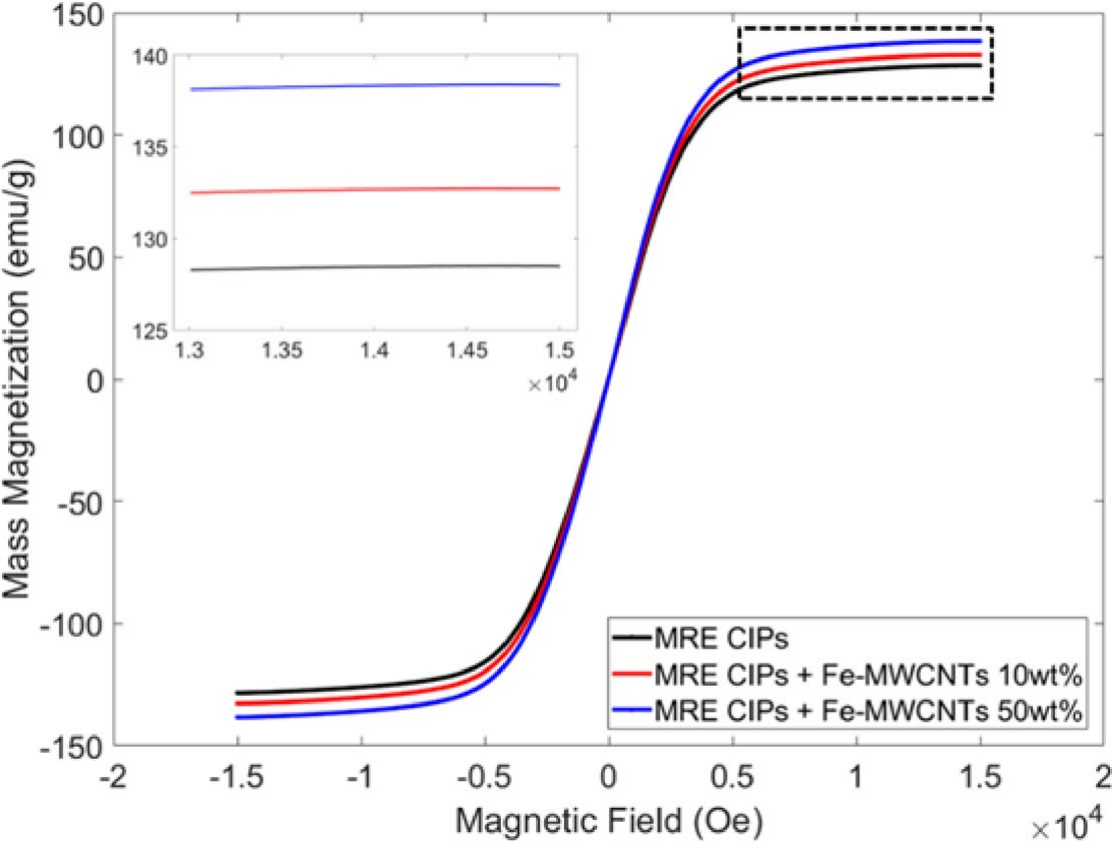
Magnetic properties of MREs.

The magnetic properties of MREs containing CIPs and Fe-MWCNTs are depicted in Fig. 9 and summarized in Table 2. The conventional MRE sample containing only CIPs showed a saturation magnetization of 128.50 emu/g. This value increased with the addition of 1 wt% of Fe-MWCNTs 10 wt%, and Fe-MWCNTs 50 wt% into the composition of CIPs-based MREs, reaching 132.72 emu/g and 138.38 emu/g, respectively. The remanent magnetization rose from 0.41 emu/g (CIPs-based MRE) to 0.45 emu/g (MRE Fe-MWCNTs 10 wt%) and 0.52 emu/g (MRE Fe-MWCNTs 50 wt%). Furthermore, coercivity that reflects the resistance to demagnetization also showed a slight increase for the CIPs-based MREs with MRE Fe-MWCNTs additive. The value increased from 11.72 Oe for the CIPs-based MRE to 12.25 Oe and 13.46 Oe for the MRE Fe-MWCNTs 10 wt% and MRE Fe-MWCNTs 50 wt%, respectively.

### Rheological properties of MREs

#### Amplitude sweep

**Fig. 10:**
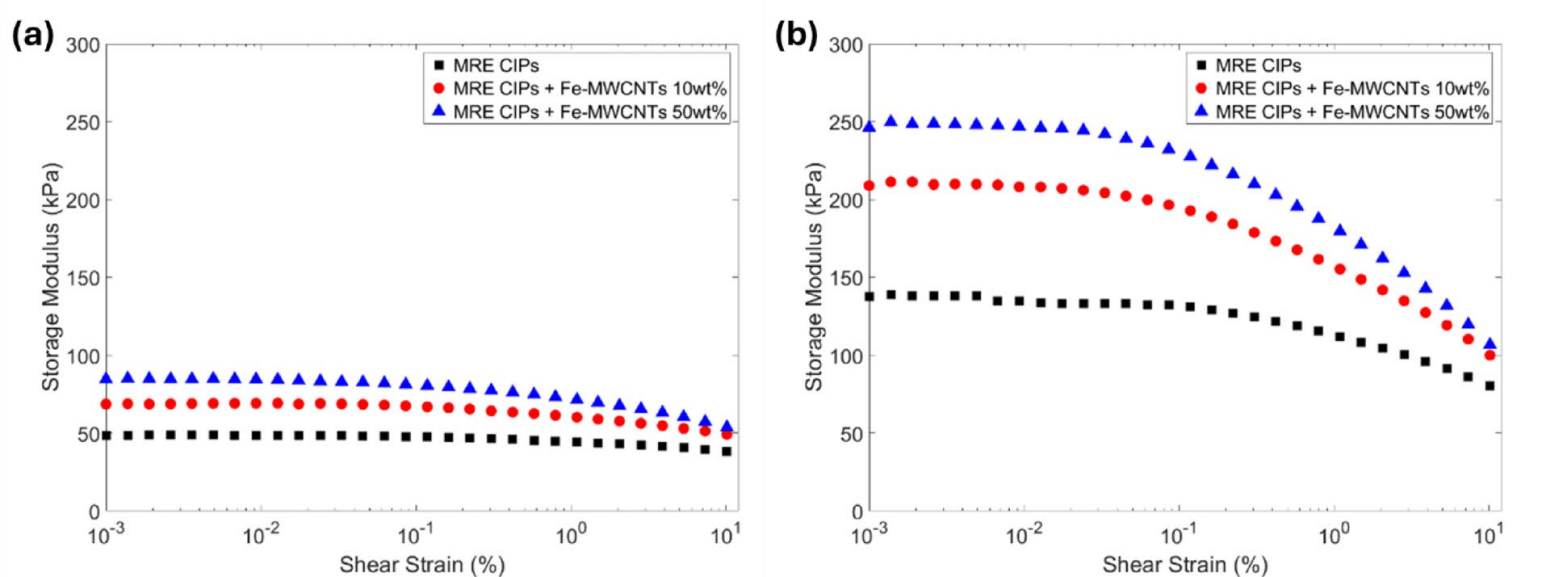
Storage modulus of MREs under amplitude sweep at different current inputs. (**a**) Current input of 0 A. (**b**) Current input of 3 A.

**Fig. 11:**
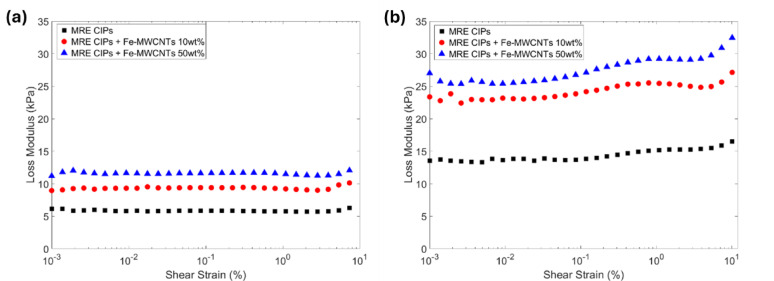
Loss modulus of MREs under amplitude sweep at different current inputs. (**a**) Current input of 0 A. (**b**) Current input of 3 A.

The amplitude sweep results of storage modulus and loss modulus of MREs can be seen in Figs. 10 and 11, respectively. This result was obtained from the rheometer test after applying different shear strains (0.001% to 10%) with the constant frequency (1 Hz) at off-state and on-state conditions (0 A and 3 A). The CIPs-based MRE was measured, showing the initial storage modulus only up to 48.433 kPa (0 A) and 137.66 kPa (3 A). Meanwhile, the initial storage modulus increased significantly with the addition of Fe-MWCNTs 50 wt% to MRE, reaching 84.68 kPa at 0 A and 246.17 kPa at 3 A, as shown in Fig. 10a and b, respectively. The loss modulus exhibits a similar trend. The measured loss modulus of CIPs-based MRE was 6.148 kPa (0 A) and 13.55 kPa (3 A), while CIPs-based MRE with the addition of Fe-MWCNTs 50 wt% reached 11.19 kPa at 0 A and 19.85 kPa at 3 A, as seen in Fig. 11a and b, respectively.

The resistance of MREs to deformation under stress can be assessed through their linear viscoelastic (LVE) behavior, which reflects how well they maintain their structure when subjected to shear strains. LVE can be defined as any point within the stable range of storage modulus, where the internal structure of the sample remains unchanged. Its storage modulus remains constant, which means the material will return to its original state after the stress is removed^51,58–60^. In contrast, when the strain amplitude surpasses this region, the materials are included in the non-linear viscoelastic (NLVE) region, characterized by a noticeable drop in storage modulus, indicating the internal structural breakdown. The LVE region of each sample at off-state condition (0 A) was around 0.222% (MRE CIPs), 0.118% (MRE CIPs + Fe-MWCNTs 10 wt%), and 0.0857% (MRE CIPs + Fe-MWCNTs 50 wt%). Meanwhile, the LVE region at on-state condition (3 A) was 0.118% (CIPs), 0.0454% (MRE CIPs + Fe-MWCNTs 10 wt%), 0.0241% (MRE CIPs + Fe-MWCNTs 50 wt%).

**Table 2:** Magnetic properties of MREs.

Materials	Magnetic saturation (emu/g)	Remanent magnetization (emu/g)	Coercivity (Oe)
MRE CIPs	128.50	0.41	11.72
MRE CIPs + Fe-MWCNTs 10 wt%	132.72	0.45	12.25
MRE CIPs + Fe-MWCNTs 50 wt%	138.38	0.52	13.46

#### Frequency sweep

**Fig. 12:**
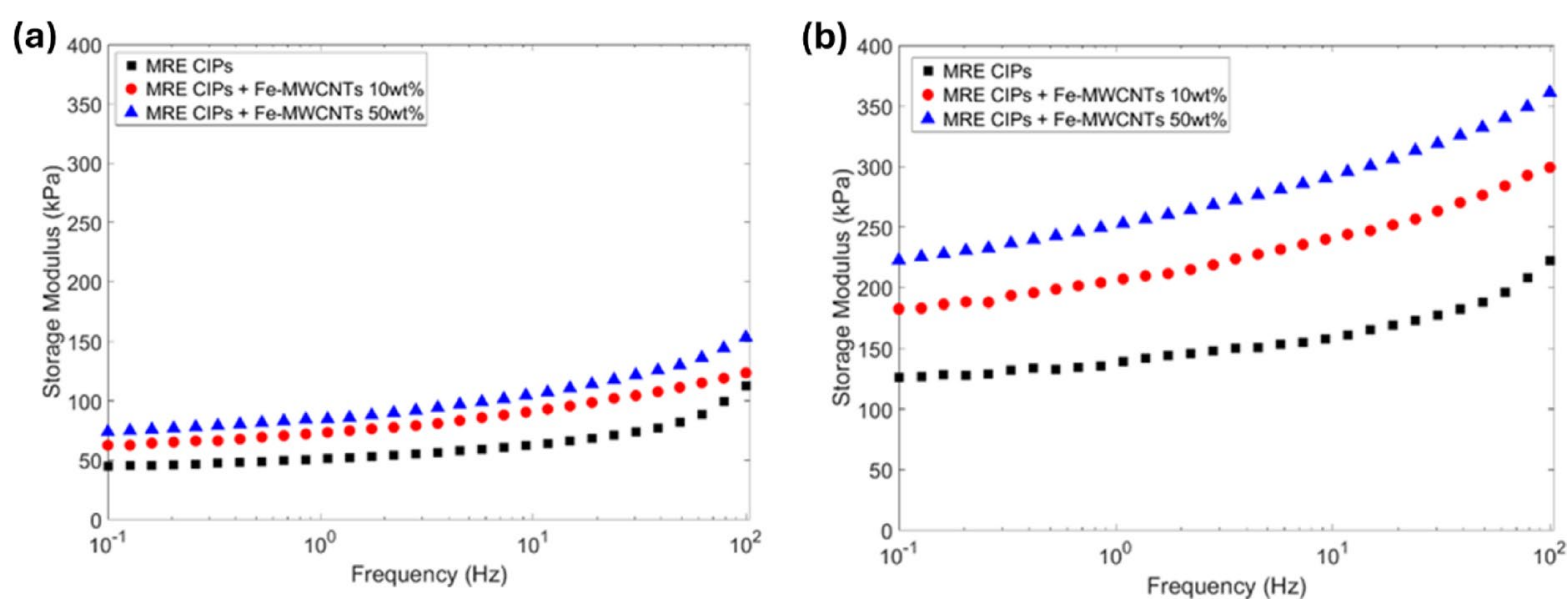
Storage modulus of MREs under frequency sweep at different current inputs. (**a**) Current input of 0 A. (**b**) Current input of 3 A.

**Fig. 13:**
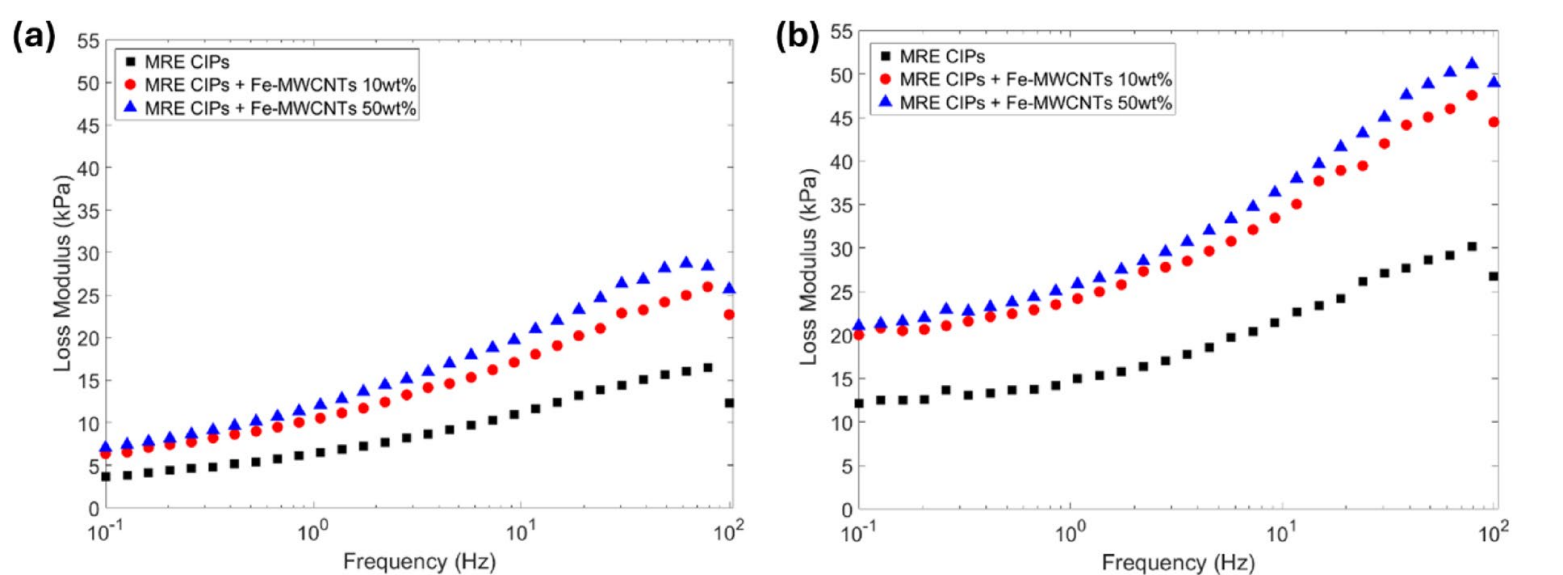
Loss modulus of MREs under frequency sweep at different current inputs. (**a**) Current input of 0 A. (**b**) Current input of 3 A.

It is crucial to conduct frequency sweep tests to evaluate the dynamic stiffness and damping behavior of MREs since they are widely utilized in vibration and acoustic applications. Both the storage modulus and the loss modulus of MREs were measured over a frequency range of 0.1 to 100 Hz with a constant strain amplitude of 0.02% under off-state (0 A) and on-state (3 A) conditions, as shown in Figs. 12 and 13, respectively.

The MRE containing only CIPs exhibited an initial storage modulus of 3.70 kPa at 0 A and increased to 12.12 kPa at 3 A, as depicted in Fig. 12a and b, respectively. Meanwhile, a significant improvement of MRE in the storage modulus was observed with the addition of Fe-MWCNTs 50 wt%, reaching 7.07 kPa at 0 A and 21.05 kPa at 3 A. The loss modulus of the CIPs-based MRE was also measured, resulting in 3.70 kPa at 0 A and 12.12 kPa at 3 A, as illustrated in Fig. 13a and b, respectively. The MRE sample with Fe-MWCNTs 50 wt% additive demonstrated higher values, reaching 7.07 kPa at 0 A and 21.05 kPa at 3 A.

#### Magnetic field sweep

**Fig. 14:**
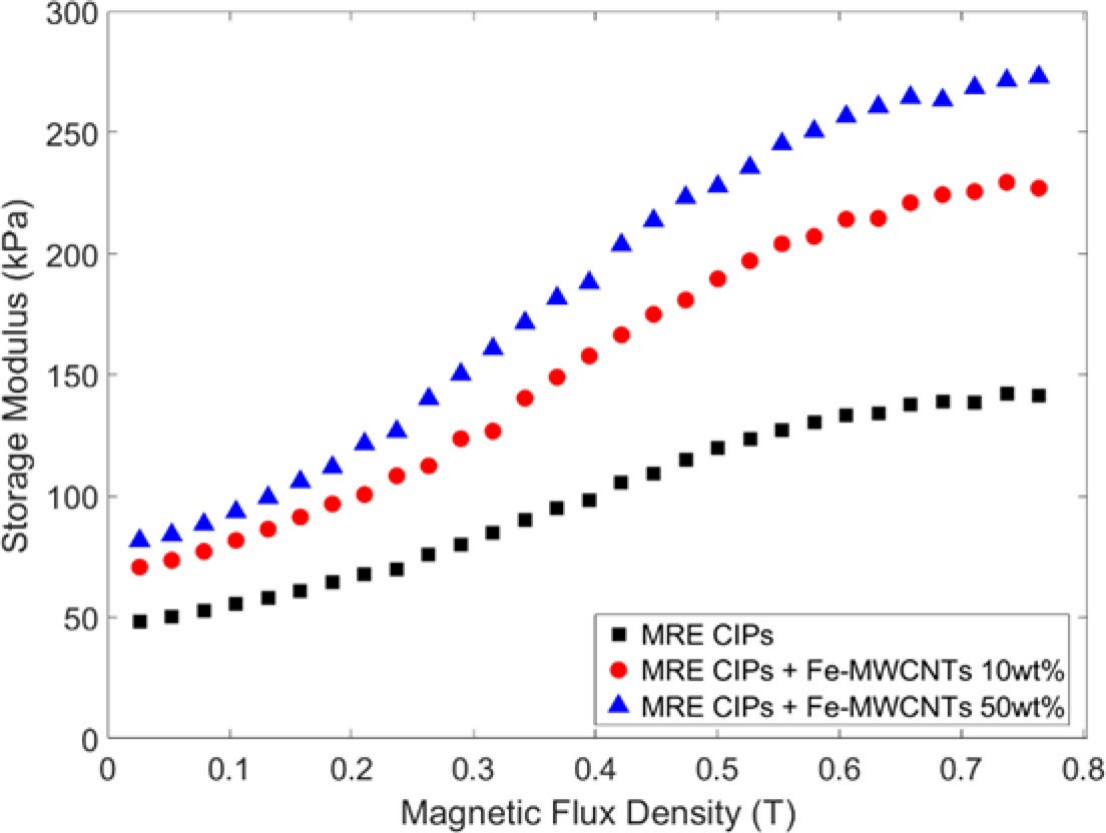
Storage modulus of MREs under magnetic field sweep.

The CIPs and Fe-MWCNTs embedded in the MRE matrix became magnetized due to dipole-dipole interactions among them when subjected to shear under an external magnetic field. This magnetization increases the energy required for the particles to move from their stable positions, leading to a shear modulus that varies with the magnetic field. Additionally, the particles tend to align along the direction of the applied magnetic field and constrain the deformation of the matrix, thereby generating its overall modulus. Therefore, magnetic field sweep tests were conducted to investigate this phenomenon. The magnetic field sweep was set up into a constant shear strain (0.02%) and frequency (1 Hz) with the applied magnetic field up to 0.7 Tesla for the measurement of the properties. The measurement results are depicted in Fig. 14.

The MR effect is the key parameter used to quantify this field-induced change in modulus, which is defined as Eq. 1. The MR effect of the MREs is summarized in Table 3. The MR effect of CIPs-based MRE, CIPs + Fe-MWCNTs 10 wt% MRE, and CIPs + Fe-MWCNTs 50 wt% MRE were 191%, 220%, and 234%, respectively. The MR effect of CIPs-based MRE (conventional MRE) was the lowest compared to the ones that were added with Fe-MWCNTs 10 wt% and Fe-MWCNTs 50 wt%, indicating a weaker filler-matrix interaction of CIPs-based MRE.

**Table 3:** MR effect of MREs.

Materials	Initial storage modulus (G_0_) (kPa)	Absolute MR effect (∆G) (kPa)	MR effect (%)
MRE CIPs	48.39	92.90	191
MRE CIPs + Fe-MWCNTs 10 wt%	70.73	156.28	220
MRE CIPs + Fe-MWCNTs 50 wt%	81.64	191.31	234

## Discussion

The morphological structures of the additives play a crucial role in enhancing the overall properties of the MRE. According to TEM and EDS results, as seen in Figs. 4 and 5, the hollow rod-like morphology and the presence of multiple carbon layers confirmed the structure of MWCNTs. Meanwhile, Fe nanoparticles were found to be uniformly dispersed and attached to the surfaces of the MWCNTs. Such morphological structures of MWCNTs and the existence of Fe on the surface of MWCNTs are believed to facilitate stronger interfacial interactions and improved interconnection between the elastomer matrix and CIPs, thereby contributing to the enhanced performance of the MREs.

The magnetic properties of Fe-MWCNTs were measured using VSM to investigate the role of Fe-functionalized carbon nanotubes in enhancing the magneto-responsive behavior of the MREs. According to the magnetic properties results in Fig. 7, it is confirmed that CIPs provide superior magnetic strength and responsiveness, indicated by their low coercivity and remanent magnetization. These soft magnetic properties are ideal for the composition of MREs, offering their characteristics to be easily magnetized and demagnetized. In addition, it was found that the addition of higher Fe doping on MWCNTs offers additional magnetic properties (magnetic saturation, coercivity, and remanent magnetization). The uniform dispersion and Fe nanoparticles within the MWCNTs may prevent aggregation, strengthen dipolar interactions, stabilize magnetic domains, and eventually lead to the rise of the magnetic properties’ values. Similar findings of the magnetic properties of MWCNTs filled with rod-shaped iron nanoparticles and magnetite coated iron nanoparticles were also reported in previous studies by Sameera et al. and Adi et al., respectively^61,62^. Overall, based on the relationship between mass magnetization (M) and magnetic field (H) curve, all particles exhibited narrow hysteresis loops, indicating that the materials efficiently respond to external magnetic fields.

This study aims to investigate the properties of MRE, thereby verifying overall structure of the MRE is essential to ensure successful fabrication. According to HR-FESEM images as seen in Fig. 8, MRE samples were successfully fabricated indicated by the evenly distributed CIPs and MWCNTs within the matrix. This observation was also supported by the results of EDS mapping, showing the existence of silicon (Si), carbon (C), and iron (Fe). In addition, magnetic characterization of MREs (Fig. 9) was also done to investigate the compatibility and interfacial interaction between the Fe-MWCNT fillers and the elastomer, as uniform and stable magnetic behavior indicates good dispersion and strong interfacial bonding in the MREs. It can be noted that the addition of Fe-MWCNTs to the CIPs-based MREs enhances the magnetic properties of the MREs, suggesting better performance in magnetically responsive applications, particularly under higher magnetic field strengths, due to the increased value of magnetic saturation. The observed rise in remanent magnetization and coercivity suggests improved magnetic retention and resistance to demagnetization, which can be advantageous for applications requiring stability in magnetic behavior. This steady increase suggests that the Fe-MWCNTs contribute additional magnetic content and enhance the overall magnetization response of the MREs. Although the increase was not significant, it highlights a better and stronger interaction between the Fe-MWCNTs and the magnetic particles within the silicone rubber matrix.

The mechanical behavior of MREs is characterized by two key viscoelastic parameters, which are the storage modulus and the loss modulus. Storage modulus reflects the elastic response of the material, representing its ability to store mechanical energy and indicating the overall stiffness of the MREs. In contrast, the loss modulus quantifies the viscous response, corresponding to the energy dissipated as heat during cyclic deformation and thus describes the material’s damping capability. The results of the amplitude sweep using the rheometer can be seen in Figs. 10 and 11. The results show that a higher amount of Fe doping content on MWCNTs increased the storage modulus and loss modulus, offering a wide range of field-dependent characteristics of MREs in stiffness and damping behavior. In addition, when a stronger magnetic field was applied to the MREs, their storage modulus and loss modulus increased. This occurs as the magnetic particles reorient and align into structured chains as the magnetic field direction, leading to a stiffer and higher-damping capability. The LVE region of the MREs was also analyzed in the amplitude sweep. According to Marzuki et al.^63^, the stiffness of a material can be identified from the length of its LVE region. A shorter LVE regions indicate a higher stiffness. It can be noted that the addition of Fe-MWCNTs and higher applied magnetic fields exhibits a shorter LVE region, indicating stiffened characteristics.

In addition, the frequency sweep results of MREs are shown in Figs. 12 and 13. The storage modulus of MRE samples increased steadily as frequency increased, regardless of whether the magnetic field was applied. This trend refers to typical characteristics of viscoelastic materials, where higher frequencies result in greater molecular entanglement. At elevated frequencies, polymer chains are unable to respond quickly enough to the applied shear force, thereby increasing the storage modulus of MREs (stiffness). Meanwhile, in accordance with the influence of the magnetic field, the dissipated energy of MREs at off-state conditions is lower compared to on-state conditions. It can also be noted that a higher doping content of Fe on the MWCNTs as the additive of MREs showed a higher loss modulus, showing that more energy is converted to heat during the deformation. This is associated with the viscoelastic response of the MREs, where the material’s internal friction converts mechanical energy into heat. Materials with viscoelastic behavior, such as MREs, act partly as springs and partly as dashpots. They exhibit intermediate phase angles along with energy dissipation under cyclic loading. The dissipated energy converts to heat, resulting in a temperature rise in rubber materials. Theoretically, the heat generated per unit volume per cycle can be described by the correlation shown in Eq. (2) as follows^64,65^.

2 $$\:Q=\varDelta\:E.f$$

where *Q* is the heat generation rate, *∆E* is the energy dissipation that represents the loss modulus, and *f* is the frequency of loading oscillation. This shows that the total heat generated per unit time is proportional to the amount of dissipated energy in each loading cycle, multiplied by how frequently the cycles occur. Therefore, an increase in the dissipated energy per cycle or in the frequency results in greater heat generation. In addition, the higher dissipated energy indicates a higher damping capacity for MREs with higher doping content of Fe-MWCNTs compared with the CIPs-based MRE. Unlike the previous study proposed by Aziz et al.^42^ using pure MWCNTs as an additive of MREs, the damping capacity of the samples showed slightly lower values compared to the conventional MRE (without MWCNTs) and had nearly identical loss factor, indicating no notable improvement in damping behavior. This may occur since the matrix is filled with particles, and the non-magnetic nature of MWCNTs restricts the mobility between the particles, thereby decreasing energy dissipation and overall damping performance.

As seen in the magnetic field sweep (Fig. 14), it shows that the storage modulus of MREs steadily increased with the magnetic fields. In addition, the MREs with Fe-MWCNTs additive showed a higher storage modulus compared with the CIPs-based MRE due to a stronger interaction between particles and matrix. Furthermore, as seen in Table 3, the addition of Fe-MWCNTs into MRE resulted in a higher MR effect. This shows that a higher Fe doping content contributed to the improvement of the alignment between particles, which strengthened their bonding, exhibiting the enhancement of controllable field-dependent characteristics. In comparison with previous studies, the obtained MR effect is considerably higher than those reported for other carbon-based additive^34^ as our work improves the MR effect approximately up to 234% by only adding 1 wt% of Fe-MWCNTs 50 wt% into the MRE. For instance, Chen et al.^38^ demonstrated that adding 7 wt% carbon black into anisotropic natural rubber-based MREs enhanced the MR effect to 104%, while isotropic silicone-based MREs with carbon black exhibited only a 22% improvement^39^. Similarly, Li et al.^41^ achieved an MR effect enhancement of 70% with 1 wt% pure MWCNTs. Aziz et al.^42^ observed only a 17.5% improvement with functionalized MWCNTs. Furthermore, the study also noted a reduction in damping performance due to the non-magnetic nature of CNTs, and an excessive content of COOH-MWCNTs (> 1.5 wt%) even diminished the MR effect as the alignment of CIPs was hindered.

**Fig. 15:**
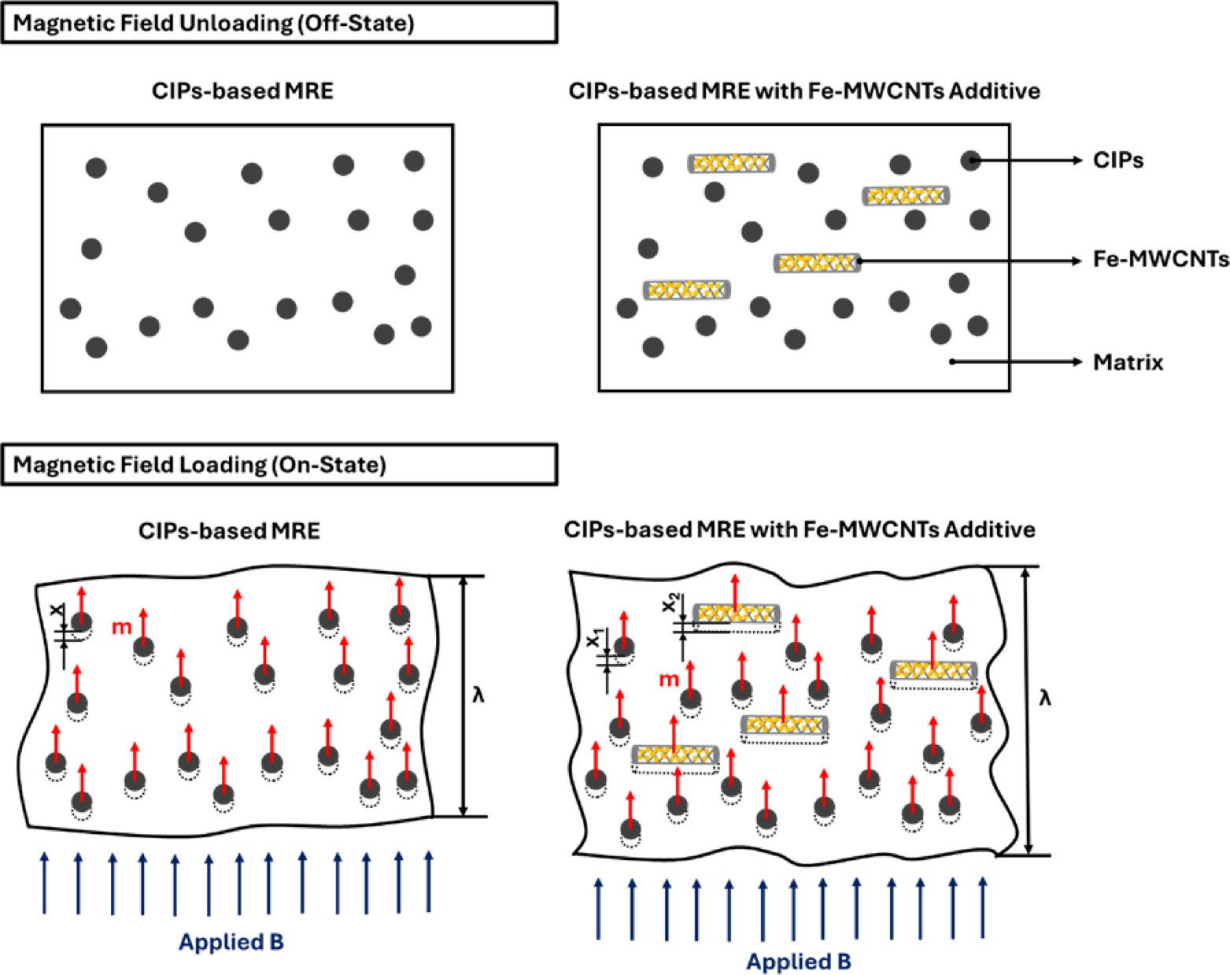
Particles interaction within the matrix.

Overall, the obtained viscoelastic behaviors of MREs are related to their interaction of the particles within the matrix that is more likely as seen in Fig. 15. The MREs with the addition of Fe-MWCNTs exhibit lower resistance to deformation under the applied magnetic field as the presence of magnetization (*m*) of CIPs and Fe-MWCNTs, leading to a greater displacement of the maparticles, denoted as *x*_*1*_ and *x*_*2*_. The induced magnetization of the CIPs, combined with the elongated hollow morphology of MWCNTs with Fe nanoparticles attached to their surface, strengthens inter-particle coupling and facilitates more effective formation and alignment under an applied magnetic field. In contrast, conventional CIP-based MREs generate magnetization only on CIPs, resulting in higher resistance to deformation in the on-state condition and comparatively smaller displacement, denoted as *x*. This effect is associated with magnetostriction^66^. When a magnetic field is applied to an MRE, particles align and form chains, causing them to change shape due to the magnetically induced stretch (λ). The larger displacements *x*_*1*_ and *x*_*2*_ compared to *x* reflect the increased mobility and reconfiguration of particles in the MRE with Fe-MWCNTs additives. As a result, MREs containing Fe-MWCNTs experience more significant internal structural changes under magnetic fields, leading to enhanced magnetically induced responses. This enhancement is evident in both the storage modulus and loss modulus, reflecting improved stiffness and damping behavior, respectively.

## Conclusion

This research investigates the effect of the addition of nanoparticles, specifically Fe-MWCNTs 10 wt% and Fe-MWCNTs 50 wt% as additives into CIPs-based MREs. The samples were then compared with the conventional MRE (CIP-based MRE). The findings of this study can be summarized as follows:

- TEM images and EDS showed the attachment of the Fe element as the doping particles on the surface of the MWCNTs. The dispersion of CIPs (spherical shape) and Fe-MWCNTs (rod-like shape) within the silicone rubber was then confirmed using HR-FESEM and EDS, showing that the MREs were successfully fabricated.
- The magnetic properties of CIPs were measured using VSM, indicating the soft magnetic characteristics of the particles. In addition, the magnetic properties of Fe-MWCNTs increased with the increasing doping content of Fe.
- The enhancement of rheological properties, both storage modulus (stiffness) and loss modulus (damping capacity), was obtained with CIPs-based MRE by incorporating Fe-MWCNTs with a higher doping composition.
- The addition of Fe-MWCNTs exhibited stronger particle interactions throughout the matrix, contributing to the improvement of the rheological properties and MR effect. The MRE containing 50 wt% Fe-MWCNTs exhibited the highest MR effect (234%), followed by the MRE with 10 wt% Fe-MWCNTs (220%), while the conventional CIPs-based MRE showed the lowest value (191%).

Overall, the results indicate that Fe-MWCNTs can effectively enhance the field-dependent rheological behavior of MREs. While this suggests potential relevance for applications requiring tunable stiffness or damping, particularly in vibration-damping systems such as dampers, earthquake resistance, machinery isolators, and engine mounts, which are typically made of elastomeric materials (rubber-like elastomeric composites) to absorb and control vibration. This work is limited by the lack of long-term stability analysis, which is necessary to understand the material’s performance over extended operational periods such as temperature and environmental aging. Additionally, since this study focused on isotropic MREs with a single matrix concentration (1 wt%), future work will explore anisotropic MREs and a broader range of additive concentrations to optimize material properties for specific application requirements. Such efforts will be essential for identifying materials with the appropriate properties for specific application requirements.

## Data Availability

The datasets used and/or analyzed during the current study are available from the corresponding author onreasonable request.

## Abbreviations

CIPs: Carbonyl iron particles
CNTs: Carbon nanotubes
COOH-MWCNTs: Carboxylic multi-walled carbon nanotubes
EDS: X-ray spectroscopy
Fe-MWCNTs: Iron-doped multi-walled carbon nanotubes
h-BNNPs: Hexagonal-boron nitride nanoplatelets
HDPE: High-density polyethylene
HR-FESEM: High-resolution field emission scanning electron microscopy
LVE: Linear viscoelastic region
MR: Magnetorheological
MREs: Magnetorheological elastomers
MRFs: Magnetorheological fluids
MWCNTs: Multi-walled carbon nanotubes
Mxene: Ti_3_C_2_T_x_
NLVE: Non-linear viscoelastic
NPTs: Non-pneumatic tires
OH-MWCNTs: Hydroxylic multi-walled carbon nanotubes
PU: Thermos-responsive polyurethane
rHDPE: Recycled high-density polyethylene
SiC: Spherical-shaped shaped silicon carbide
SMPNC: Shape memory polymer nanocomposite
SMPs: Shape memory polymers
TEM: Transmission electron microscopy
VSM: Vibrating sample magnetometer
XRD: X-ray diffraction
